# Melanogenesis inhibitory effect of aerial part of *Pueraria thunbergiana* in vitro and in vivo

**DOI:** 10.1007/s00403-014-1489-z

**Published:** 2014-07-26

**Authors:** EunByeol Han, BoYoon Chang, DaeSung Kim, HyoungKwon Cho, SungYeon Kim

**Affiliations:** 1 Institute of Pharmaceutical Research and Development, College of Pharmacy, Wonkwang University, 460 Ikandae-ro, Iksan, 570-749 Jeollabuk-do Republic of Korea; 2 Hanpoong Pharm. CO., Ltd, 333-24 1st Palbok-dong, Deokjin-gu, Jeonju, 561-841 Jeonbuk Republic of Korea

**Keywords:** *Pueraria thunbergiana*, Anti-melanogenesis, GSK-3β, Glucosidase, In vivo

## Abstract

Melanin is major factor that determines skin color as well as one of the defense systems that prevent the UV-induced damage. In case of abnormal concentration of melanin, skin diseases or problems occur such as albinism, leukoplakia, melasma, freckles, moles, and lentigo. With the lifespan of humans has been extended, importance of ‘life quality’ has been increased. White and clean skin is very important part of the satisfaction of appearance, especially for Asia women. The aim of this study was to find an anti-melanogenesis activity for which the aerial part of *Pueraria thunbergiana* can be utilized based on the increase in demands for cosmetics, particularly natural products. We demonstrated anti-pigmentation effects of aerial part of *P. thunbergiana* by measuring melanin content and through staining in the B16F10 melanoma cell line. The aerial part of *P. thunbergiana* decreased tyrosinase activity significantly in B16F10 cell cultures, while there is no direct effect on enzyme in cell-free conditions. To define the mechanisms, real-time PCR, western blot, glucosidase activity and antioxidant activity assay were implemented. As results, we demonstrated that aerial part of *P. thunbergiana* has anti-melanogenesis activity via two mechanisms. One is downgrading microphthalmia-associated transcription factor by activating Akt/GSK-3β. Consequently, transcription of tyrosinase and tyrosinase-related protein 1 is decreased. Another is interrupting maturation of tyrosinase through inhibiting α-glucosidase. Furthermore, aerial part of *P. thunbergiana* showed great efficacy on pigmentation in vivo. These results suggest that aerial part of *P. thunbergiana* can be used as an anti-melanogenic agent.

## Introduction

*Pueraria thunbergiana*, also known as the kudzu, is a species of climbing plant belonging to the Leguminosae family. The root and flower of *P. thunbergiana*, used in traditional medicine, have various medicinal properties [15, 33, 35, 36, 38]. However, the vine of *P. thunbergiana* is commonly discarded as a waste product and presents an environmental problem. The vine grows by climbing adjoining structures and trees, and destroys forests and landscape because of its weight and fast rate of growth. In some countries, *P. thunbergiana* is considered among the invasive species and seen as a threat to the ecosystem, with its management exacting a high cost, both financially and in terms of manpower [9, 16].

Melanin is a major factor that determines skin color, as well as one of the defense systems that prevent UV-induced skin damage. Abnormal concentrations of melanin manifest as skin diseases or problems, such as albinism, leukoplakia, melasma, freckles, moles, and lentigo. Skin-whitening agents are commonly applied for treating pigmentation and pigmentary diseases. Because of an increasing interest in herbs, many studies focused on discovering novel natural skin-whitening agents that are currently underway [30]. We investigated, therefore, whether the aerial part of *P. thunbergiana*, which is currently considered a waste product, has potentially useful skin-whitening effects.

Melanin is synthesized in melanocytes by enzyme-related mechanism, such as tyrosinase, tyrosinase-related protein 1 (TRP1) and tyrosinase-related protein 2 (TRP2). The first step of melanogenesis is a transformation of l-tyrosine into 3,4-dihydroxyphenylalanine (l-DOPA), and this l-DOPA is converted into DOPA quinine [28]. Then from this product, melanin is synthesized by subsequent transformation. First two ensuing stages, regulated by tyrosinase, are rate-limiting steps because the rest of the melanin synthesis process can go along naturally at a physiological pH [11]. Since tyrosinase is the key enzyme of melanogenesis, many whitening agents are targeting the tyrosinase by various mechanisms such as interference with tyrosinase catalytic activity directly, inhibition of mRNA expression, interruption of tyrosinase maturation, and acceleration of tyrosinase degradation [1].

Transcription levels of melanogenic genes are regulated by microphthalmia-associated transcription factor (MITF) [32]. MITF is expressed by that kind of stimulating factors. On the other hand, mitogen-activated protein kinase (MEK)/extracellular signal-regulated kinases (ERK) and Akt/glycogen synthase kinase-3β (GSK-3β) signals pathways are involved in the down-regulation of melanogenesis by phosphorylating MITF [18, 34]. The phosphorylated MITF decreases its activity, binding to tyrosinase promoter site, and eventually degrades. Thereby, activation of MEK/ERK or Akt/GSK-3 inhibits melanin synthesis [14, 18].

The melanogenesis is regulated by nutritional and hormonal factors. l-tyrosine and l-DOPA are not only substrates but also bioregulator for melanogenesis. Regulation for melanocyte function of l-tyrosinase and l-DOPA can be mediated through specific receptors or without receptors [28]. In addition, there are complex multiple hormonal stimulators such as MSH, adrenocorticotropic hormone (ACTH), endorphin and stem cell factor (SCF) and inhibitors such as serotonin, melatonin, dopamine, acetylcholine and melanin-concentrating hormone (MCH) [25].

Since in vivo study is also important regarding the transmembrane or transdermal permeation, it was undertaken using cream formulations. There are differences between epidermal and follicular melanogenesis although basic features such as existence of melanocyte and melanosome are common. The follicular melanogenesis depends on anagen stage of hair cycle, whereas epidermal melanogenesis is continuous and follicular melanocyte is more influenced by aging [26]. Consequently, melanin-generating hairless mice irradiated UVB were used for in vivo study [31]. When skin exposes to UV, α-melanocyte-stimulating hormone (α-MSH), one of the important hormones which modulate pigmentation, is produced as a response [5].

## Materials and methods

### Reagents

Antibiotic–antimycotic, bovine serum albumin (BSA), Dulbecco’s modified Eagle’s medium (DMEM), and fetal bovine serum were purchased from Life Technologies (Carlsbad, CA, USA). Acarbose, dimethyl sulfoxide (DMSO), 3,4-dihydroxy-l-phenylalanine (l-DOPA), 2,2-diphenyl-1-picrylhydrazyl (DPPH), ethylenediaminetetraacetic acid (EDTA), α-glucosidase (EC 3.2.1.20, from baker’s yeast, 77 U/mg), LY294002, α-melanocyte-stimulating hormone (α-MSH), mushroom tyrosinase and *ρ*-Nitrophenyl-α-d-glucopyranoside were purchased from Sigma-Aldrich (St. Louis, MO, USA). Fontana–Masson staining kit was obtained from IHCWORLD (Woodstock, MD, USA). Phosphate-buffered saline (PBS) was obtained from Bioworld Technology, Inc. (BioWorld, USA). easy-Blue™ Total RNA extraction kit was purchased from Intron Biotechnology, Inc. (iNtRON Biotechnology, Seongnam, Korea). TaqMan^®^ RNA-to-Ct™ 1-Step Kits were purchased from Applied Biosystems (USA). RIPA buffer was obtained from Biosesang Inc. (Seongnam, Korea). Protease inhibitor cocktail tablets were purchased from Roche Diagnostics (Switzerland). Antibodies were purchased from Cell Signaling Technology (Danvers, MA, USA) and Santa Cruz Biotechnology Inc. (Santa Cruz, CA, USA). ECL reagent was purchased from GE Healthcare.

### Extraction of plant material

Aerial parts of *P. thunbergiana* were collected from Jinan, Jeonbuk, Korea, in November 2010, and extracted by the Hanpoong Pharm and Foods Company (Hanpoong Pharm. CO., Ltd.). Briefly, dried and pulverized materials (2 kg) were boiled with 2 L of distilled water and a range of ethanol concentrations (0, 30, 70, and 95 %) for 3 h. The solvent was then removed under reduced pressure in a rotary evaporator (N-1000S, EYELA, Japan) to yield a water extract (439.5 g), 30 % ethanolic extract (409.2 g), 70 % ethanolic extract (436.7 g), and a 95 % ethanolic extract (284.8 g).

**Fig. 1:**
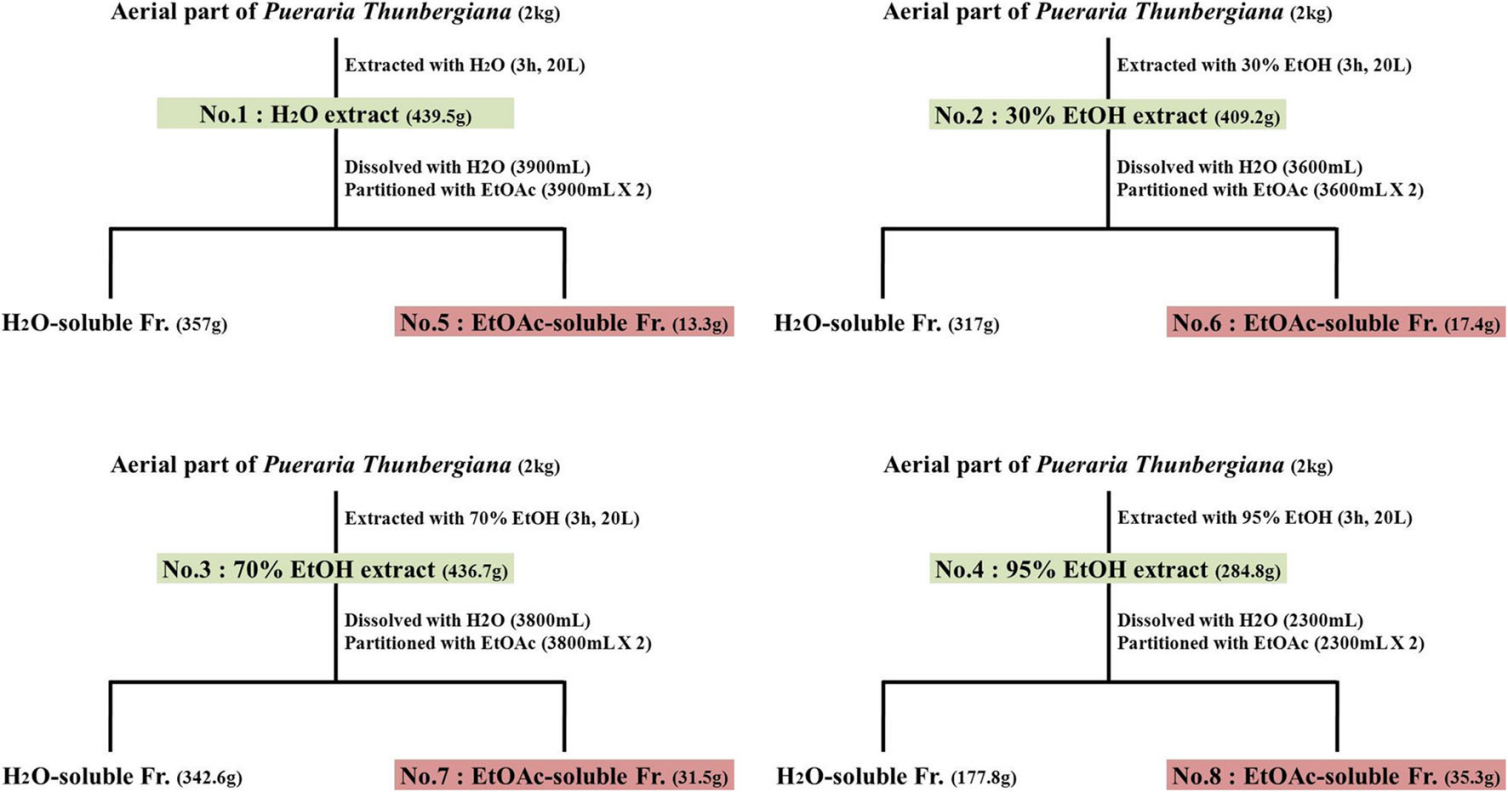
Extraction and partition of fractions from aerial part of *P. thunbergiana*

The respective extracts were suspended with distilled water, and then partitioned with ethyl acetate (EtOAc). The EtOAc and aqueous fractions were independently evaporated under reduced pressure at 60 °C, and the extracts completely dried. Aqueous fractions, with increasing ethanol concentration in the initial extraction step, are referred to as extract Nos. 1–4, while the organic (EtOAc) fraction extracts are referred to as Nos. 5–8 (Fig. 1).

### High-performance liquid chromatography analysis

**Fig. 2:**
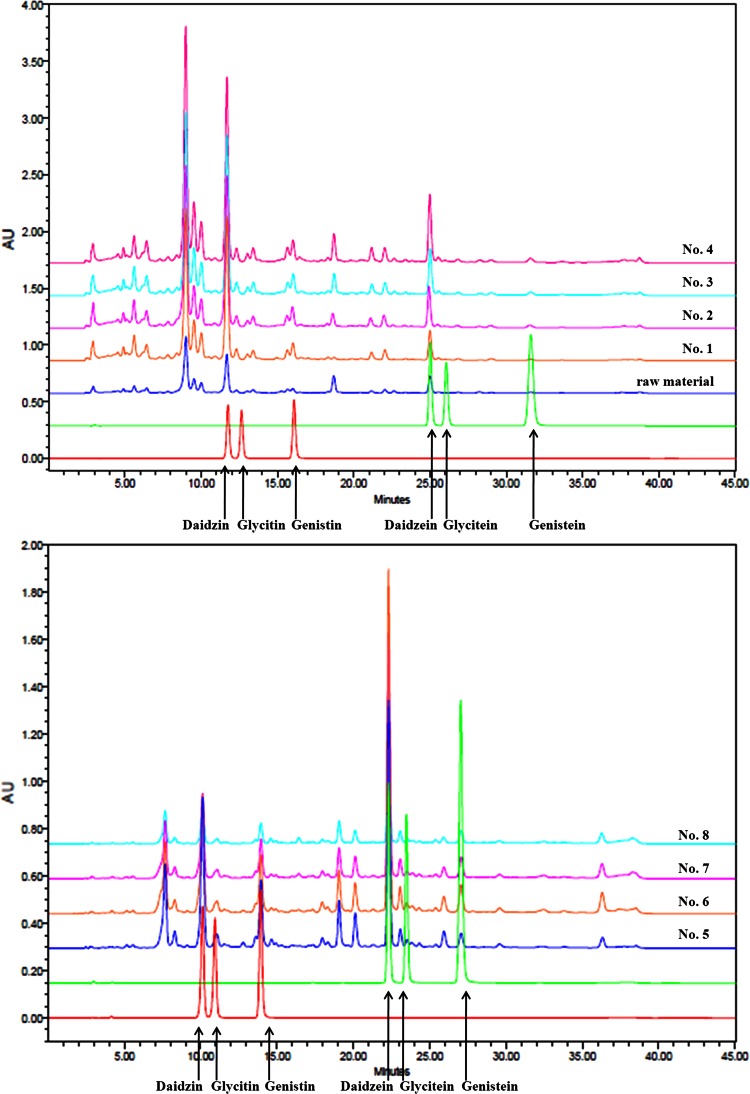
HPLC chromatogram showing peaks corresponding to isoflavones in extracts of the aerial part of *P. thunbergiana*, with absorbance evaluated at 260 nm

**Fig. 3:**
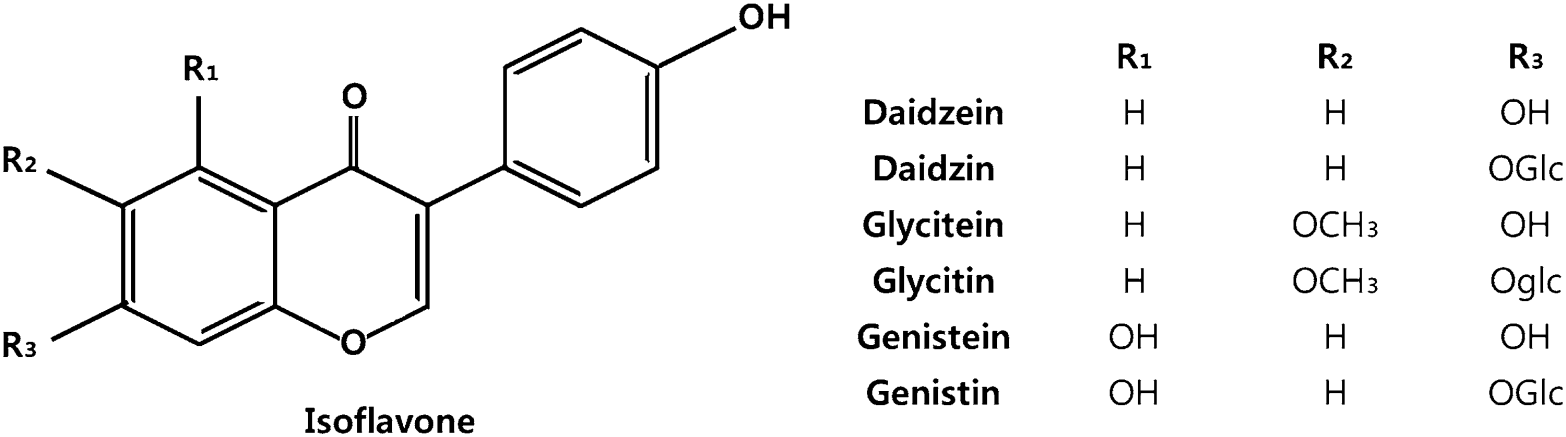
Chemical structure of isoflavones in extracts of the aerial part of *P. thunbergiana*

The isoflavone content of the extracts was quantitated by high-performance liquid chromatography (HPLC; Waters 2695 system, Waters Co., Milford, MA, USA) using a CAPCELLPAK UG 120 250 × 4.6 mm, 5-µm column (Shiseido Co., Tokyo, Japan). Separation and quantification of isoflavones were achieved at 30 °C using a solvent gradient from solution A, a 10 % aqueous methanol solution with 2 % acetic acid, to solution B, a 98 % aqueous methanol solution with 2 % acetic acid, at a flow rate of 1 mL/min. Peaks were detected by measuring absorbance at 260 nm. The presence of isoflavones was confirmed by comparing the observed peaks with the retention time of the corresponding standards (Wako Chemical Co., Osaka, Japan). The chromatogram of total isoflavones is shown in Fig. 2, and the chemical structures of each isoflavone are indicated in Fig. 3. The isoflavone aglycone, and glycoside concentrations in each extract are listed in Table 1.

**Table 1:** Composition of isoflavones from the aerial part of *P. thunbergiana*

Aerial part of *P. thunbergiana*	Aglycone (mg/g)			Glycoside (mg/g)		
	Daidzein	Glycitein	Genistein	Daidzin	Glycitin	Genistin
Raw material	1.76	0.03	0.12	6.51	0.41	0.47
No. 1	6.27	0.27	0.29	45.74	3.45	3.33
No. 2	9.22	0.39	0.60	55.04	3.83	4.33
No. 3	10.14	0.33	0.71	53.12	3.90	4.26
No. 4	14.97	0.56	1.00	61.38	4.52	4.50
No. 5	114.69	3.94	5.76	125.64	14.80	349.91
No. 6	149.59	4.52	10.33	98.24	12.44	286.61
No. 7	93.61	2.77	6.82	61.83	8.13	162.36
No. 8	78.15	2.52	5.09	37.84	5.28	101.16

### Cell culture and treatment with extracts

Murine B16F10 melanoma cells purchased from ATCC (Manassas, VA, USA) were cultured in DMEM with 10 % (v/v) fetal bovine serum and 1 % (v/v) antibiotic–antimycotic. The cells were incubated in a humidified incubator under 5 % CO_2_ at 37 °C. Aqueous fractions are dissolved in DDW and EtOAc fractions are dissolved in DMSO. Controls of B16F10 cells were added DDW or DMSO at the same concentration as in the treated cells.

### Cell viability assay

Cell proliferation was evaluated by performing the MTT assay. The cells (3 × 10^3^ cells/well) were seeded into a 96-well plate and incubated for 24 h; the cells were subsequently exposed to the *P. thunbergiana* extracts at a range of concentrations (0, 5, 10, 50, 100, 500, and 1,000 μg/mL) for 2 days. Thereafter, serum-free MTT medium was added to each well to the final concentration of 1 mg/mL, and incubation performed for 2–4 h at 37 °C. The MTT medium was removed from the wells, and DMSO was added; thereafter, the plate was placed on a shaker for 5 min. Absorbance was read at 540 nm using a microplate spectrophotometer (SpectraMax 190, USA).

### Melanin content

The B16F10 cells (3 × 10^4^ cells/well) were seeded in a 6-well plate and exposed to α-MSH (100 nM) for 1 day. The treatment was performed with a combination of α-MSH (100 nM) and *P. thunbergiana* extracts for 2 days. The cells were harvested by trypsinization and washed twice with PBS and alcohol. 2 × 10^5^ cells were dissolved in 200 μL of 1 N NaOH with 10 % DMSO at 90 °C for 1 h. The resulting melanin concentration was quantified by measuring the absorbance at 475 nm.

### Mushroom tyrosinase activity

Reactions were performed in potassium phosphate buffer (pH 6.81). Mushroom tyrosinase solution was prepared by dissolving 25,000 units in 6 mL of 0.1 mM potassium phosphate buffer and adding 2 mL of distilled water. l-DOPA solution (0.01 %) in distilled water was used as the enzyme substrate. A mixture of 160 μL of buffer, 20 μL of substrate, 10 μL of *P. thunbergiana* extract, and 10 μL of enzyme was added to a 96-well plate. Tyrosinase activity was quantified by measuring absorbance at 475 nm after 2 min. Kojic acid was used as a positive control. The data are expressed as mean ± SD.

$${\text{Tyrosinase activity }}\left( {\text{\%}} \right) = \frac{{{\text{sample OD}}_{475} }}{{{\text{control OD}}_{475} }}\; \times \;100$$

### Cellular tyrosinase assay

Another method of measuring tyrosinase activity was repeated since there are complex regulations of tyrosinase activation [24]. The B16F10 cells were exposed to α-MSH (100 nM) for 1 day and treated with a combination of extracts and α-MSH for 2 days. The pellet was obtained by trypsinization and washed with PBS. The cells were lysed with 0.1 M sodium phosphate buffer (pH 6.8) containing 5 mM EDTA, 1 % Triton X-100, and 0.1 % phenylmethylsulfonyl fluoride (PMSF) in ice for 30 min. After centrifugation of the lysate at 15,000 rpm for 30 min at 4 °C, cellular tyrosinase activity was measured in the resulting supernatant. Enzyme activity was normalized to protein concentration, as determined by Bradford assay. The cellular tyrosinase and 0.1 % l-DOPA reaction were performed in 0.1 M sodium phosphate buffer at 37 °C for 1 h. Tyrosinase activity was quantified by measuring the absorbance at 475 nm.

### Real-time PCR

Quantification of selected genes transcript by real-time PCR was performed using a TaqMan^®^ RNA-to-Ct™ 1-Step Kit according to the manufacturer’s instructions. The mRNA was extracted with easy-Blue™ Total RNA extraction kit and quantified by Nano Drop. Relative ratio of a target gene expression was calculated with the Δ*C*_t_ method.

### Western blot analysis

After treatment with a range of concentrations (0, 10, 50, and 100 μg/mL) of extracts, the B16F10 cells were washed with PBS, and lysed by RIPA buffer in ice to get the protein. Next, 20–50 μg of proteins was subjected to electrophoresis on 10–15 % sodium dodecyl sulfate-polyacrylamide gels (SDS-PAGE). Following electrophoresis, the proteins were transferred to nitrocellulose membranes, and the membranes were blocked with 5 % skimmed milk or 5 % BSA in PBS with 0.1 % Tween 20 (PBST). Then, the proteins were probed using antibodies. The immunoblots were developed and visualized using an enhanced chemiluminescence (ECL) detection system (Amersham Biosciences, Piscataway, NJ, USA). The western blot was imaged using the Chemi Documentation Imaging System and quantified using densitometric program (Image J). GAPDH and β-actin were used as an internal control.

### Glucosidase activity assay

Inhibition activities of α-glucosidase were evaluated according to the chromogenic method described by McCue et al. [17]. Yeast α-glucosidase (0.5 unit/mL) 20 µL, 0.1 M phosphate buffer (pH 6.9) 120 and 10 µL of test sample dissolved in DMSO at various concentrations were mixed in a microplate well. After incubating for 15 min at 37 °C, 20 µL of *ρ*-Nitrophenyl-α-d-glucopyranoside (5 mM) was added to substrates and incubated for an additional 15 min. The reaction was terminated by 80 µL of 0.2 M sodium carbonate (Na_2_CO_3_) solution. The absorbance was measured at 405 nm. Acarbose was used as positive control. Each experiment was conducted in triplicate, and the IC_50_ values of samples were calculated.

### Antioxidant activity

DPPH was dissolved in methanol to a 0.1 mM concentration, and mixed with the diluted *P.* *thunbergiana* extracts (0, 10, 50, and 100 μg/mL) in a 96-well plate in a 1:1 ratio. Reaction was allowed to proceed in the dark for 30 min, and the absorbance was measured at 520 nm. Ascorbic acid was used as a standard. The data are expressed as mean ± SD.

### Animals

Six-week-old male hairless mice, Hos: HRM2, were purchased from Hoshino Laboratory Animals Inc. (Yashio, Saitama, Japan). The animals were housed in a SPF animal facility room at 23 ± 1 °C and 50 ± 10 % relative humidity with 12-h light/dark cycle with free access to standard commercial diet and water. Creams containing 1 or 3 % of No.6 extracts were used for assessing in vivo anti-melanogenesis efficacy. There are five groups of mice in the animal model; control group (applied cream base, CON), UVB-exposed control group (UVB + applied cream base, UVB), positive control group (UVB + applied 1 % kojic acid cream, kojic acid), experiment group 1 [UVB + applied 1 % No.6 cream, No.6 (1 %)] and experiment group 2 [UVB + applied 3 % No.6 cream, No.6 (3 %)].

### In vivo skin pigmentation determinations

The treatments were performed once per every day since 3 days before UVB irradiation by topically applying 100 mg of each cream on the dorsal skin of the animal. The mice were anesthetized with a ketamine–xylazine mixture (2 mL/kg, i.p., respectively) and after 30 min to absorb the cream, exposed to UVB at 100, 150 and 200 mJ/cm^2^ once per day for each 5 days, respectively. Colors of skin were determined using a DSM II ColorMeter (Cortes Technology). The results were expressed as L* values (value of 0 indicates black, 100 indicates white). At the 19 day, mice were sacrificed and the dorsal skins were obtained. The specimens were used for Fontana–Masson staining. ΔL* were calculated as follows:

$${{\Delta }}L^{ *} = \left( {\frac{{{\text{day}}\;2 + {\text{day}}\; 3 + {\text{day}}\;4}}{3}} \right) - \left( {\frac{{{\text{day}}\;17 + {\text{day}}\;18 + {\text{day}}\;19}}{3}} \right)$$

### Fontana–Masson staining

The B16F10 melanoma cells were cultured and treated in a chamber slide. After removal of the medium, the cells were fixed with 4 % formalin for 20 min and washed twice by PBS containing 0.1 % Triton X-100. The cells were stained with the Fontana–Masson staining kit according to manufacturer’s instructions.

Each specimen was soaked in formaldehyde to fix. Then, paraffin processing and embedding were carried out. The tissue blocks were sectioned at 4 μm. The skins were stained with Fontana–Masson staining kit.

Briefly, the B16F10 cells or specimens were incubated in pre-warmed FM silver nitrate solution, toned in gold chloride solution, and soaked in 5 % sodium thiosulfate solution. Finally, counterstaining was performed with the Nuclear Fast Red Solution.

### Statistical analysis

All results obtained from at least three independent experiments were combined and analyzed using the Student’s *t* test. All values were expressed as the mean ± SD. The acceptable level of significance was established at *p* values <0.05

## Results

### The aerial part of *P. thunbergiana* hinders melanogenesis in B16F10 cells

**Fig. 4:**
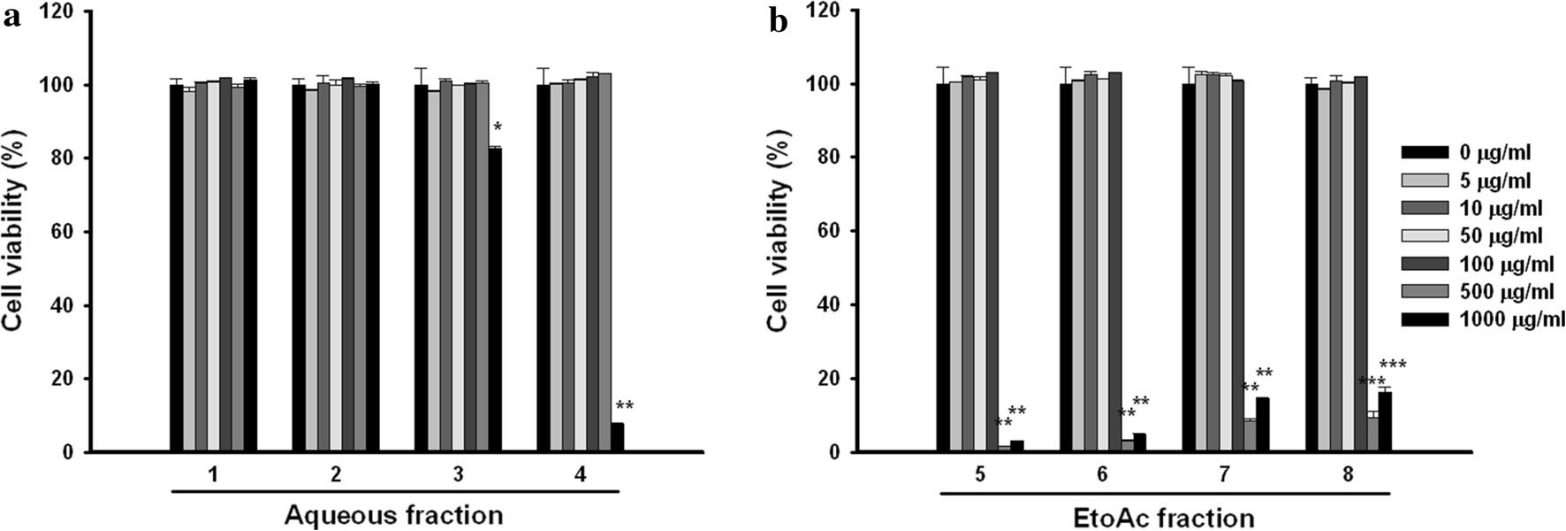
Concentration-dependent effects of aerial part of *P. thunbergiana* on B16F10 cell viability. Cytotoxicity of *P. thunbergiana* extracts in B16F10 melanoma cells was evaluated by treating the cells treated with aqueous or EtOAc fraction of extracts for 48 h. Cell viability was determined using the MTT assays, and results are expressed as percent viability relative to untreated cells

**Fig. 5:**
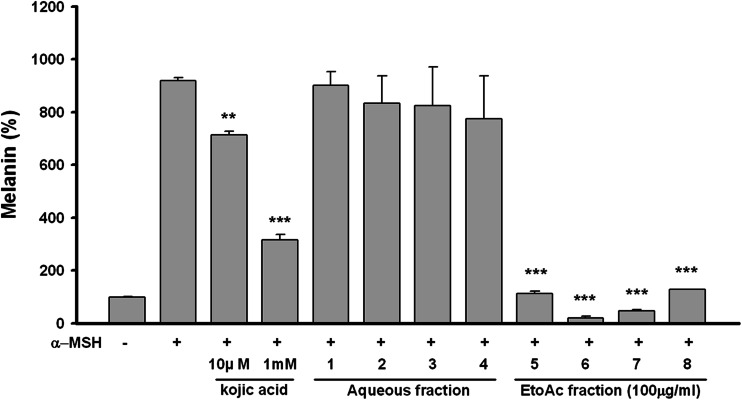
Effect of aerial part of *P. thunbergiana* on melanogenesis in B16F10 cells. B16F10 cells were cultured, and following adherence, the cells were treated with α-MSH. After 1 day, the cells were incubated with combinations of α-MSH and kojic acid or *P. thunbergiana* extract Nos. 1–8. Melanogenesis was quantified 2 days later, and the results expressed relative to controls. **p < 0.01, and ***p < 0.001 compared to cells stimulated with α-MSH alone

To evaluate the whitening effect of the aerial part of *P. thunbergiana*, B16F10 melanoma cells were treated with the extracts at a range of concentrations that showed no adverse effects on cell viability (Fig. 4). Melanin synthesis was induced by α-MSH in B16F10 cells and 100 μg/mL, the highest concentration without cytotoxicity, of samples were treated for screening. α-MSH is one of the factors that stimulate pigmentation in mammals [12]. The aqueous fractions of *P. thunbergiana* extracts had no effects on melanogenesis, but the EtOAc-soluble fractions of the extracts inhibited α-MSH-induced melanin synthesis more effectively than did kojic acid, a whitening agent used as a positive control (Fig. 5). When the data are expressed as a percentage normalized to the non-treated cells, the cells treated with extracts No.6 (EtOAc-soluble fraction of the 30 % EtOH extract), and No.7 (EtOAc-soluble fraction of the 30 % EtOH extract) showed 21.17 and 48.20 % of melanin contents, respectively, lower than that shown by the non-treated cells.

**Fig. 6:**
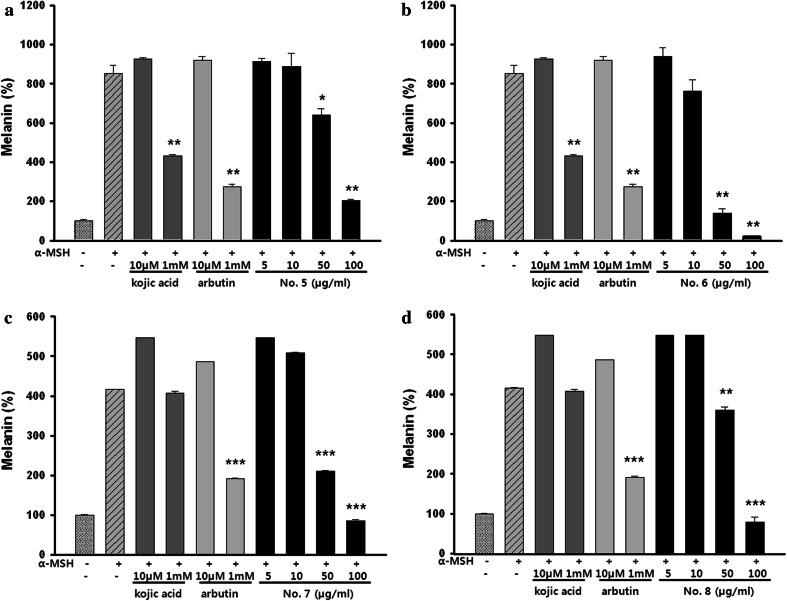
Effect of EtOAc fractions on melanogenesis in B16F10 cells. B16F10 cells were cultured, and following adherence, the cells were treated with α-MSH. The cells were treated with a combination of α-MSH with kojic acid, arbutin, or a range of concentrations of extract Nos. 5–8 (5, 10, 50, and 100 μg/mL). The results were expressed relative to untreated cells. *p < 0.05, **p < 0.01, and ***p < 0.001 compared to α-MSH-stimulated cells

**Fig. 7:**
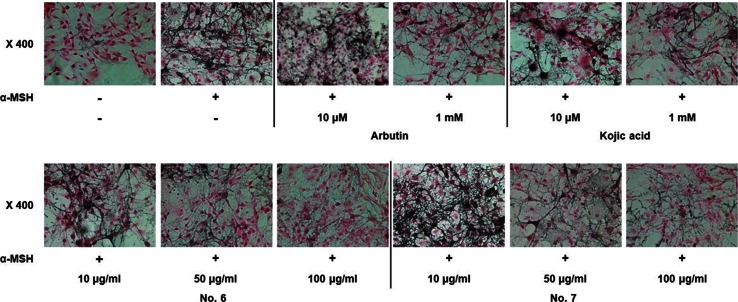
Optical images (magnification ×400) showing melanin content with Fontana–Masson staining in B16F10 cells. After 24 h of α-MSH pre-incubation, B16F10 cells were treated with a combination of α-MSH and extract No. 6 or 7, arbutin, or kojic acid for 48 h. Pigmentation observed upon Fontana–Masson staining (magnification ×400)

Further examination showed that the treatment of the cells with the EtOAc-soluble fraction of *P. thunbergiana* extracts reduced melanogenesis in a dose-dependent manner (Fig. 6). The melanogenesis-suppressing activity of extract Nos. 6 and 7, most significant samples in collective assessment, was confirmed visually by Fontana–Masson staining (Fig. 7). Accumulation of melanin was observed in α-MSH-treated B16F10 cells and treatment with *P. thunbergiana* extracts down-regulated the pigmentation in the cells. At the highest concentration used, extract No.6 inhibited melanogenesis to the baseline (non-treated cells) level.

### The aerial part of *P. thunbergiana* indirectly inhibits tyrosinase activity

**Fig. 8:**
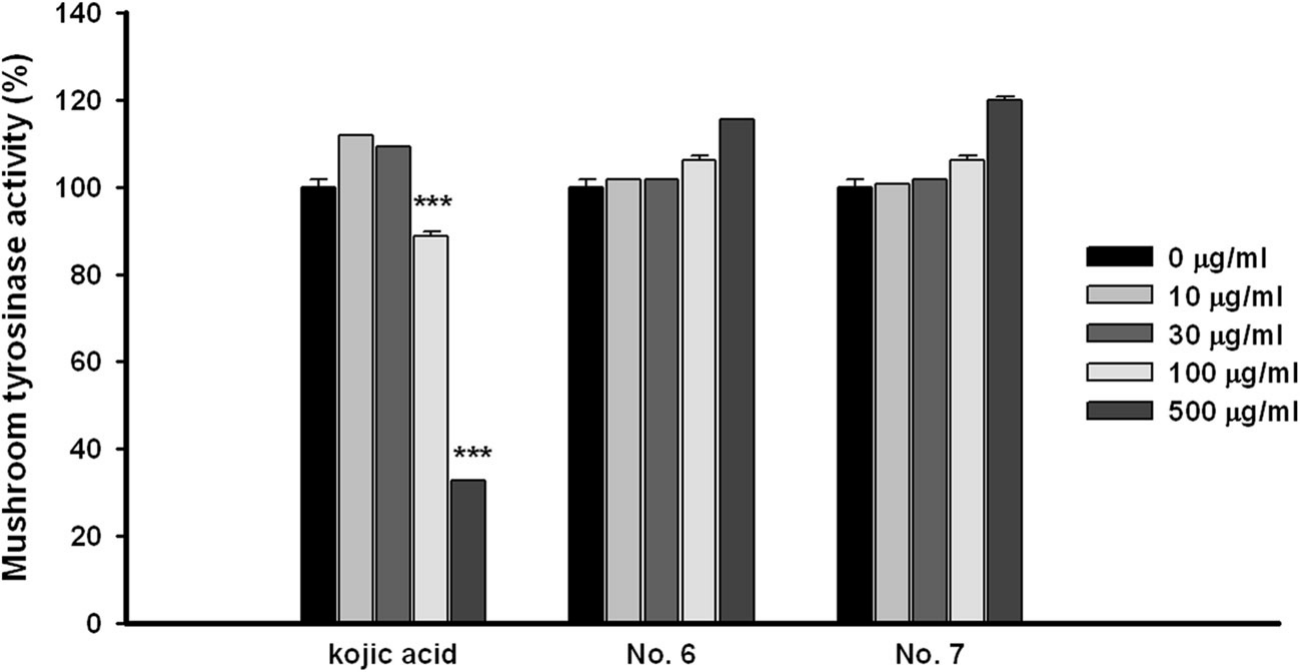
Comparison of cell-free tyrosinase activity of aerial part of *P. thunbergiana*. Reactions were performed in potassium phosphate buffer with l-DOPA and mushroom tyrosinase. After 2 min, tyrosinase activity was assessed by measuring the absorbance at 475 nm and expressed relative to the controls. Kojic acid was used as a positive control

**Fig. 9:**
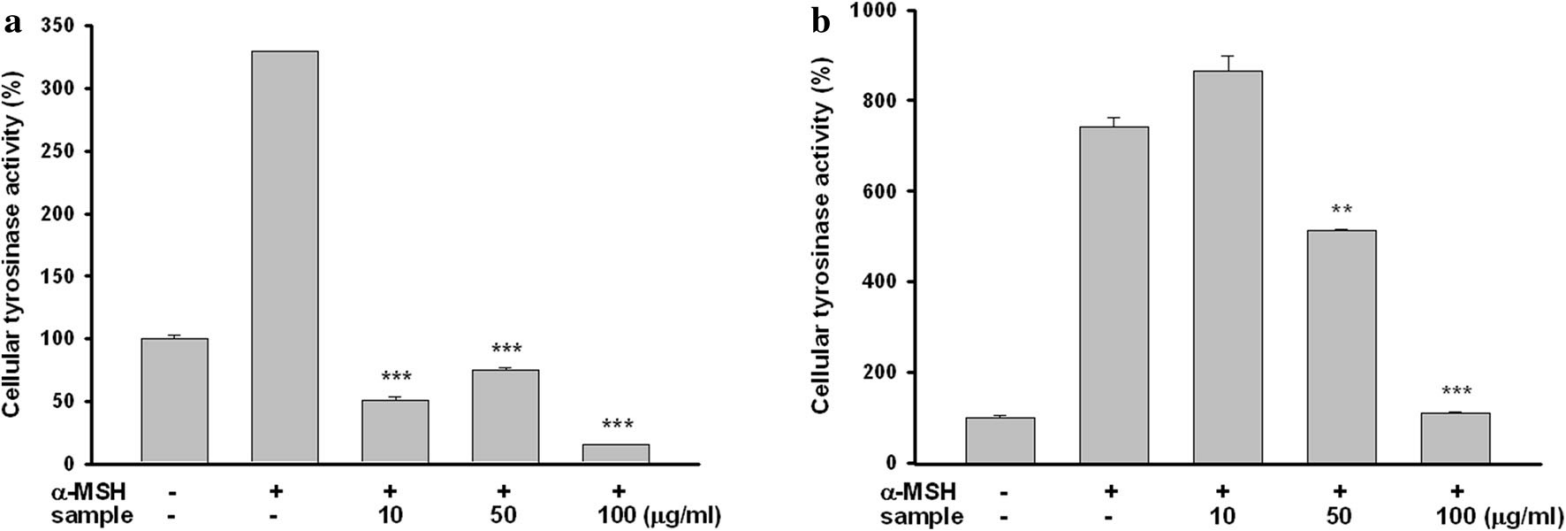
Inhibitory effects of aerial part of *P. thunbergiana* on cellular tyrosinase activity. B16F10 cells were pre-cultured with α-MSH for 24 h, and incubated for 48 h more in a medium containing several concentrations (10, 50, and 100 µg/mL) of extract No. 6 (**a**) or No. 7 (**b**). The cellular tyrosinase activity was measured and the results are expressed relative to controls. *p < 0.05, **p < 0.01, and ***p < 0.001 compared to α-MSH-stimulated cells

The process of melanogenesis is regulated by an intracellular enzymatic cascade. To determine if the aerial part of *P. thunbergiana* directly affects tyrosinase, the key enzyme for melanin synthesis, we used mushroom and cellular tyrosinase activity assays. Unlike kojic acid, *P. thunbergiana* extracts did not exhibit any inhibitory effect on the tyrosinase activity in cell-free condition (Fig. 8). Interestingly, the cellular tyrosinase in B16F10 cells stimulated by α-MSH was significantly inhibited by treatment with *P. thunbergiana* extracts in a dose-dependent manner (Fig. 9). In the case of extract No.6, cellular tyrosinase activity declined to levels lower than those in the non-treated cells (Fig. 9a).

### Effects on expression of mRNA and protein of tyrosinase

**Fig. 10:**
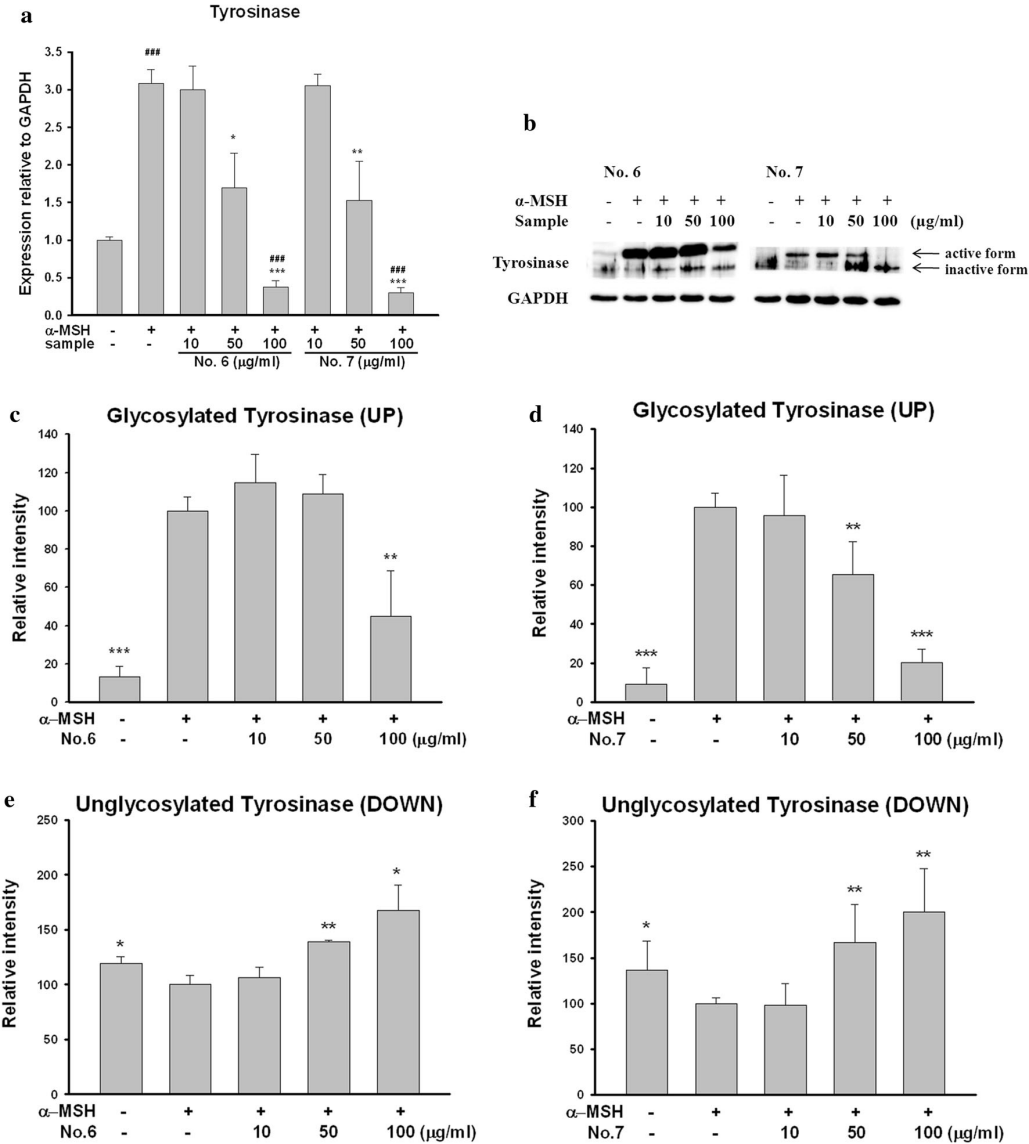
Effects of aerial part of *P. thunbergiana* on mRNA and proteins expression of tyrosinase. B16F10 cells were treated with α-MSH and No. 6 or No. 7 for the indicated concentration. mRNA levels (**a**) and expression levels (**b**) of tyrosinase were estimated. GAPDH was used as an internal standard. **c**–**f** Quantitation of the western blots by using GAPDH as the loading control. *p < 0.05, **p < 0.01, and ***p < 0.001 compared to α-MSH-stimulated cells. ^###^ *p* < 0.001 compared to nonstimulated cells

We conducted real-time PCR assay for inquiry about effects of aerial part of *P. thunbergiana* on mRNA of tyrosinase (Fig. 10a). Transcriptions of tyrosinase were significantly attenuated compared to transcription of α-MSH-stimulated cells. Moreover, we found dose-dependent manner effects of Nos. 6 and 7. Especially, tyrosinase expressions of B16F10 cells treated with 100 μg/mL were lower than the level of non-treated cells. The decrease of tyrosinase activity was further confirmed by western blot assay (Fig. 10b). There were double bands, and these bands indicate degrees of maturation of tyrosinase. Upper bands for glycosylated tyrosinase represent the mature form, while other bands for unglycosylated tyrosinase represent the immature form. Tyrosinase becomes the active form after being glycosylated. Treatment of Nos. 6 and 7 inhibited glycosylation of tyrosinase.

### Inhibitory effects on α-glucosidase

**Fig. 11:**
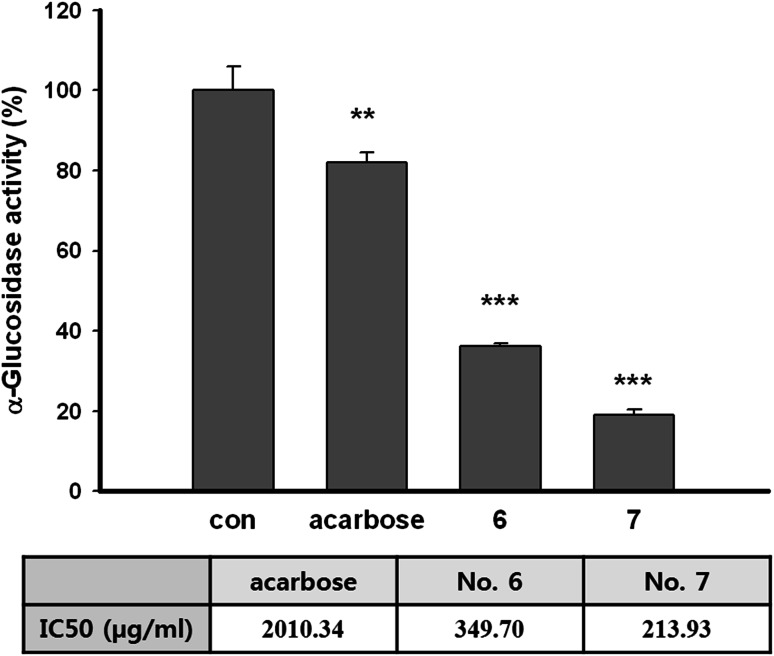
Inhibitory effects on α-glucosidase activity. α-Glucosidase was incubated with acarbose, Nos. 6, 7 (500 µg/mL) and PNPG. Each percentage value of α-glucosidase activity is reported relative to control. *p < 0.05, **p < 0.01, and ***p < 0.001 compared to control

To identify how aerial part of *P. thunbergiana* hinders maturation of tyrosinase, the study about α-glucosidase activity was conducted. Briefly, we demonstrated that both Nos. 6 and 7 have α-glucosidase inhibitory activity. The extracts inhibited α-glucosidase more effectively than acarbose, the synthetic inhibitor of α-glucosidase (Fig. 11). Dose–response curves of activity were obtained, and the IC_50_ values were calculated. No. 6 dramatically reduced α-glucosidase activity with IC_50_ of 349.70 μg/mL. The IC_50_ of No. 7 was the smallest with 213.93 μg/mL which is about ten times lower than acarbose with 2,010.34 μg/mL.

### Effects on expression of mRNA and protein of TRP1

**Fig. 12:**
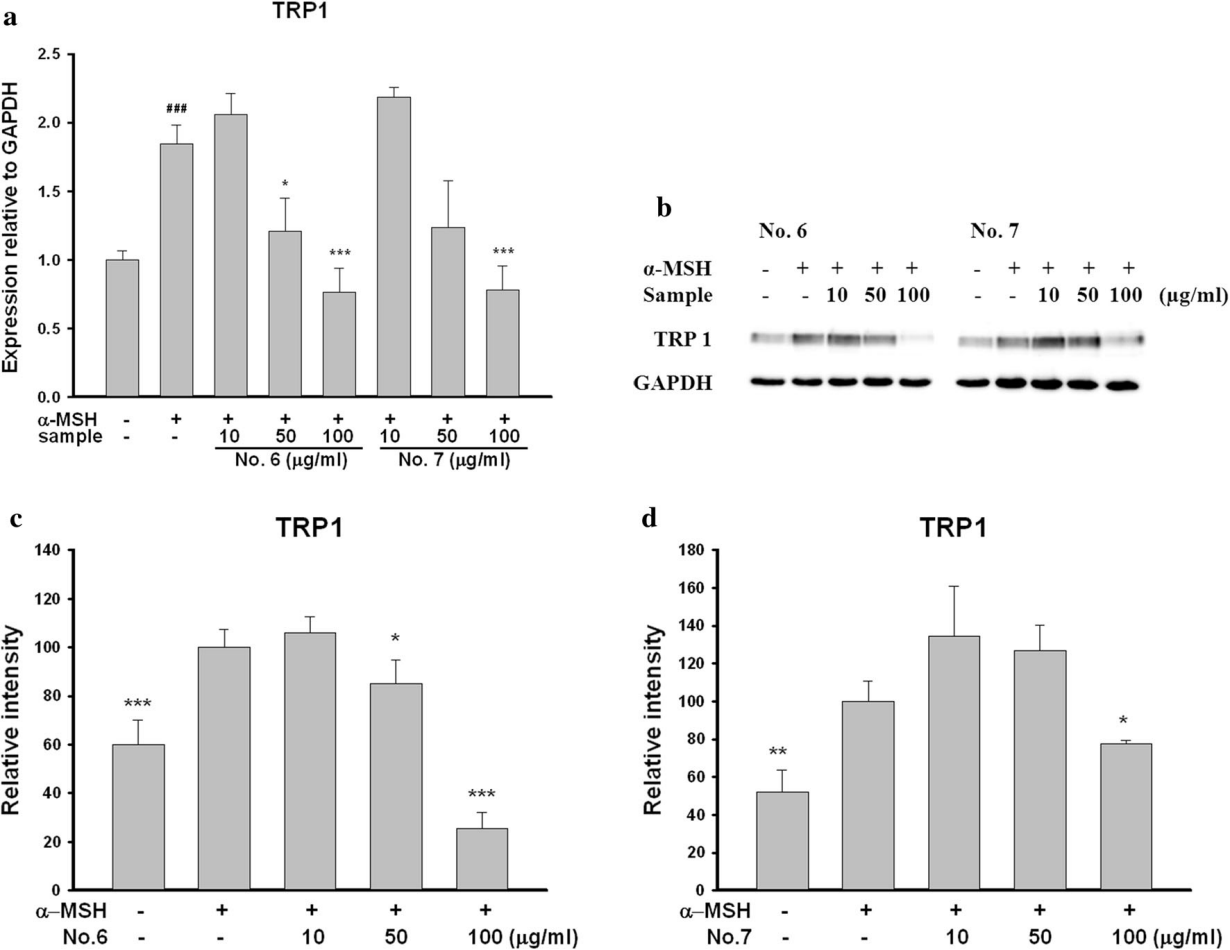
Effects of aerial part of *P. thunbergiana* on mRNA and proteins expression of TRP1. B16F10 cells were treated with α-MSH and No. 6 or No. 7 for the indicated concentration. mRNA levels (**a**) and expression levels (**b**) of TRP1 were estimated. GAPDH was used as an internal standard. **c**–**d** Quantitation of the western blots by using GAPDH as the loading control. *p < 0.05 and ***p < 0.001 compared to α-MSH-stimulated cells. ^###^ *p* < 0.001 compared to nonstimulated cells

To find out effects of aerial part of *P. thunbergiana* on TRP1, we examined the mRNA and protein levels after treating a range of concentration of Nos. 6 and 7 (0, 10, 50, and 100 μg/mL) on α-MSH-stimulated B16F10 cells. Transcriptions of TRP1 were significantly attenuated in a dose-dependent manner (Fig. 12a). Expressions of TRP1 were significantly decreased by Nos. 6 and 7 (Fig. 12b).

### Aerial part of *P. thunbergiana* inhibits MITF expression through the activation of Akt/GSK-3β signaling pathway

**Fig. 13:**
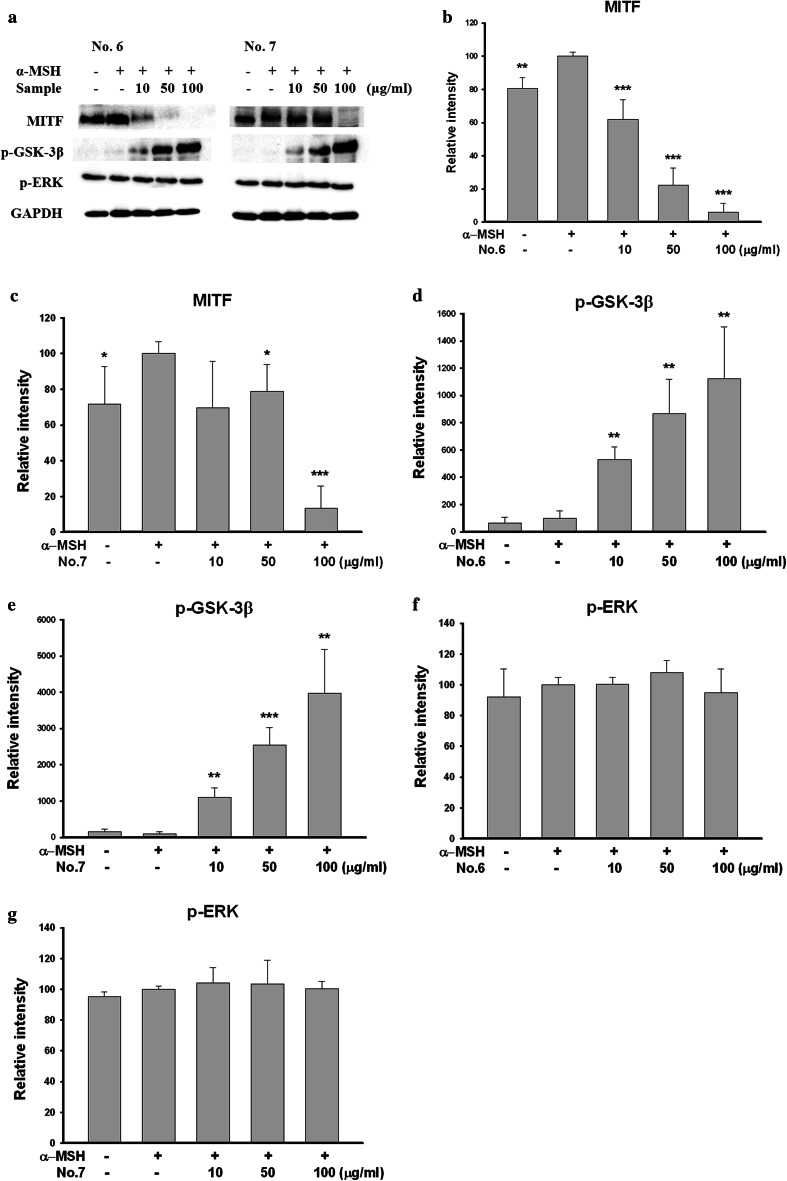
Effects of aerial part of *P. thunbergiana* on expression of melanogenesis-related proteins. B16F10 cells were treated with α-MSH and No. 6 or No. 7 for the indicated concentration. Western blot assay were performed to estimate and expression levels of total MITF, p-ERK and p-GSK-3β. Protein loading amounts were confirmed by GAPDH expression. **b**–**g** Quantitation of the western blots by using GAPDH as the loading control. *p < 0.05, **p < 0.01, and ***p < 0.001 compared to α-MSH-stimulated cells

We found that the aerial Part of *P. thunbergiana* inhibits transcription of tyrosinase and TRP1 through MITF down-regulation. As shown in Fig. 13, expression of MITF was decreased by treatments with Nos. 6 and 7. We assessed phosphorylation of GSK-3β and ERK by implementing western blot since activated Akt/GSK-3β and MEK/ERK signals degrade MITF. Phosphorylation of GSK-3β was significantly enhanced by Nos. 6 and 7, respectively. p-ERK was not significantly influenced.

**Fig. 14:**
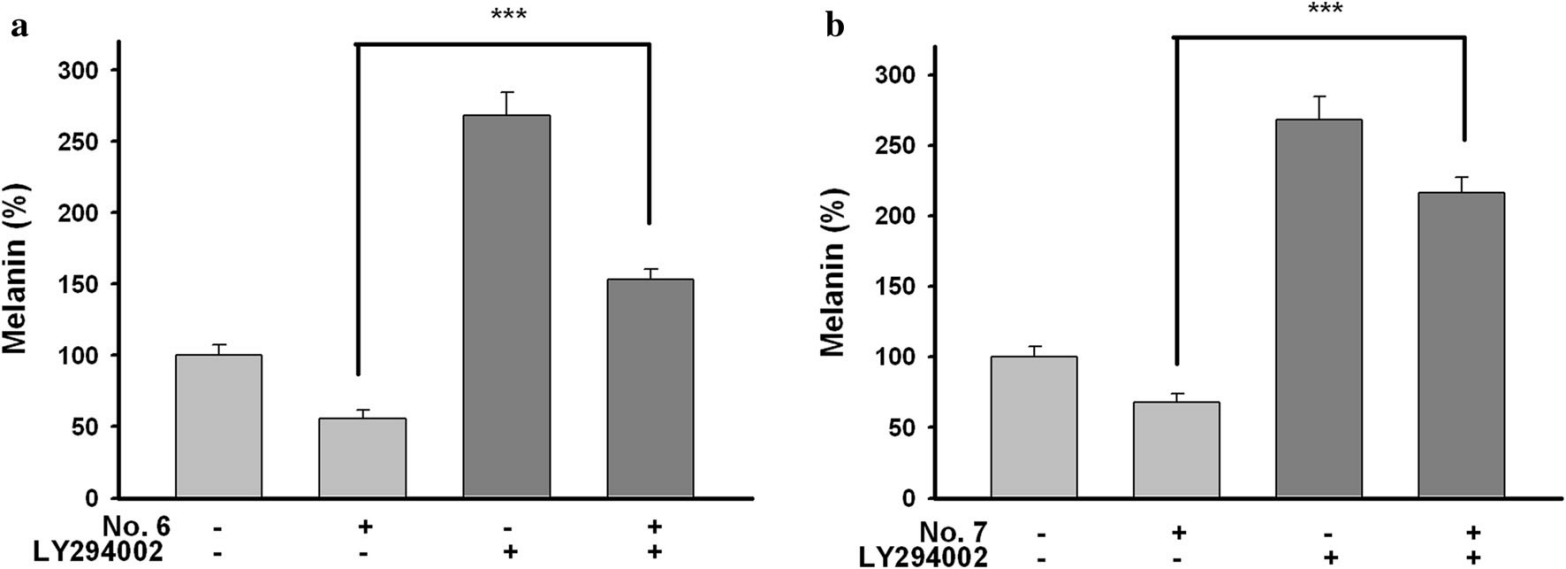
Effects of aerial part of *P. thunbergiana* on regulation of Akt/GSK-3β signal. Melanin contents were evaluated. Cells were pretreated in the absence (−) or presence (+) of LY294002 (20 µM) for 1 h and then cultured without (−) or with (+) 50 µg/mL of No. 6 (**a**) and 7 (**b**) for 48 h. ***p < 0.001 compared to sample-treated only

Therefore, we examined melanin formations in B16F10 cells co-treated with No. 6 or 7 and LY294002 as Akt/GSK-3β-specific inhibitor. As shown in Fig. 14, treatment of LY294002 induced, and treatment of Nos. 6 and 7 inhibited melanin synthesis compared with control. The melanin production, suppressed by Nos. 6 and 7, was restored by LY294002. These results suggested that the aerial part of *P. thunbergiana* activates Akt/GSK-3β.

### Evaluation of the in vivo depigmenting activity of aerial part of *P. thunbergiana*

**Fig. 15:**
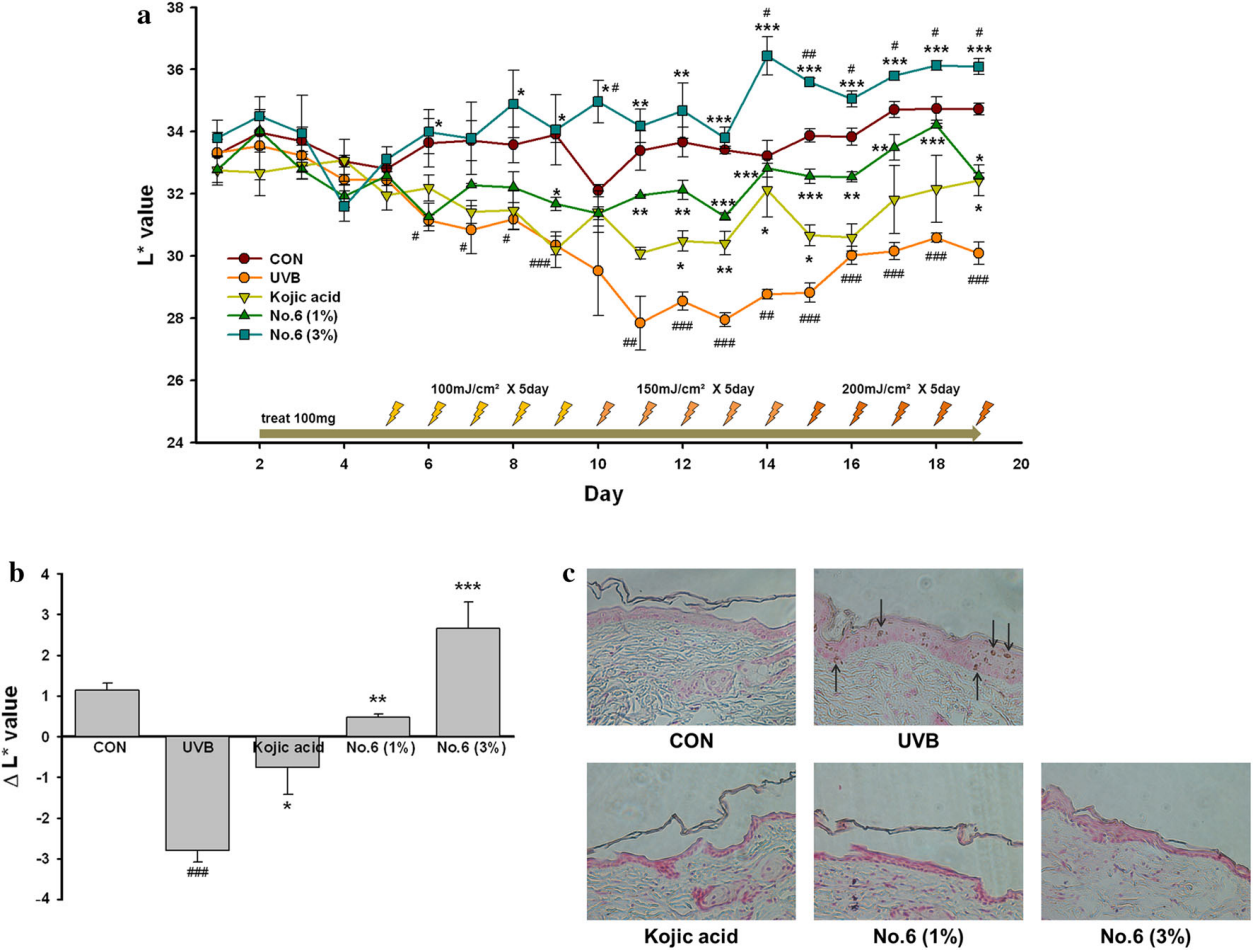
Effects of aerial part of *P. thunbergiana* on pigmentation in UV-irradiated animal. **a** Melanin possessing hairless mice were treated with 100 mg of cream base, kojic acid 1 % cream, No. 6 1 and 3 % cream on dorsal skins every day. UVB irradiation was performed according to the indicated schedules. The lightness (L* value) of skin was measured before applying cream on each day. **b** ΔL* values of each animal were calculated; values of days 17, 18, 19 minus values of days 2, 3, 4. Data represent mean ± SEM (*n* = 3). ^#^ *p* < 0.05, ^##^ *p* < 0.01, and ^###^ *p* < 0.001 compared with CON. *p < 0.05, **p < 0.01, and ***p < 0.001 compared with UVB group. **c** Microscopic images of skin sections. Melanogenesis of dorsal skin was highlighted by Fontana–Masson staining. *Arrows* indicate pigmentation

To survey the potentiality as human whitening agent, the depigmenting activity of aerial part of *P. thunbergiana* was estimated by in vivo system using melanin-procession hairless mice for 19 days. As shown in Fig. 15, the degrees of pigmentation were measured as L* value representing the lightness of the color. The values of UVB-exposed control group (UVB) have significantly decreased compared with control group (CON) since 6 day. The treatments with No. 6 creams mitigated pigmentation, respectively. The L* values were significantly higher than UVB-exposed control group (UVB) in No. 6 1 % cream-applied animals [No. 6 (1 %)] after 9 day. Besides, in No. 6 3 % cream-applied animals [No. 6 (3 %)], the values were not decreased by UVB irradiation and have been significantly higher than the values of UVB-exposed control (UVB) since 8 day. Furthermore, the values have increased at comparing with control group (CON) since 14 day (Fig. 15a). For positive control, the calculated UVB-induced decreases of L* values were alleviated by application of kojic acid 1 % cream (Kojic acid) (Fig. 15b).

### Microscopic observation of the epidermis

Effects of aerial part of *P. thunbergiana* on pigmentation in UVB-irradiated mice were confirmed by Fontana–Masson staining (Fig. 15c). In the UVB-exposed-control epidermis, an induction of melanogenesis was observed compared to controls. In mice applied with No. 6 (1 and 3 %), melanogenesis was reduced.

## Discussion

Currently, numerous compounds are used for skin whitening, such as arbutin, hydroquinone, and kojic acid. However, there is growing interest in finding alternative agents since the existing ones possess mutagenic properties and can induce skin disorders or low depigmenting activity [8, 20].We investigated, therefore, the effects of the aerial part of *P. thunbergiana*, an overlooked resource, on melanin synthesis. Both aqueous and EtOAc fractions of the extracts obtained using a range of EtOH densities (0, 30, 70, and 95 %) were studied.

We found that the EtOAc fraction significantly reduced melanin synthesis in the B16F10 melanin-processing cell lines (Fig. 5) without causing cytotoxicity (Fig. 4). The *P. thunbergiana* extracts exhibited dose-dependent inhibition, with extract Nos. 6 and 7 (30 and 70 % ethanol, respectively) proving to be excellent melanogenesis inhibitors (Fig. 6). In addition, anti-pigmentation effects of the aerial part of *P. thunbergiana* were clearly demonstrated by the intracellular melanin accumulation observed using staining techniques (Fig. 7).

A number of mechanisms exist for the regulation of melanogenesis [10]. The most common target for depigmenting agents is tyrosinase, the rate-limiting enzyme of melanogenesis. Whitening ingredients such as ascorbic acid, thio-containing compounds, phenolic compounds, and kojic acid inhibit tyrosinase enzyme activity [6]. In this study, we have established that the aerial part of *P. thunbergiana* reduces tyrosinase activity through its effect on cellular tyrosinase in α-MSH-treated B16F10 cells (Fig. 9). However, the aerial part of *P. thunbergiana* had no direct inhibitory effects on tyrosinase (Fig. 8). These results indicate that the anti-melanogenesis activity of the aerial part of *P. thunbergiana* is involved in superior levels regulating tyrosinase enzyme such as transcription, translation, and maturation [23].

To understand the mechanisms of inhibitory effects on melanogenesis, we examined the effects on mRNA and protein expression of tyrosinase and TRP1, enzymes involved in melanogenesis. Aerial part of *P. thunbergiana* inhibits transcription and protein levels of tyrosinase and TRP1 (Figs. 10, 12).

Transcription of tyrosinase and TRP1 is promoted by MITF [2, 32]. We found out that MITF was down-regulated by aerial part of *P. thunbergiana* (Fig. 13). It was reported that the activation of Akt/GSK-3β and MEK/ERK signaling pathway downgrades activity and stability of MITF and leads to degradation [13]. Accordingly, we investigated the phosphorylation of GSK-3β and ERK. Phosphorylation of GSK-3β was increased significantly by aerial part of *P. thunbergiana* in a dose-dependent manner. p-ERK had no change (Fig. 13). In addition, we found that melanin formations, which were inhibited by aerial part of *P. thunbergiana*, recovered with the Akt/GSK-3β-specific inhibitor LY294002 (Fig. 14). These results mean that aerial part of *P. thunbergiana* inhibits MITF through modulation of the Akt/GSK-3β pathway, not MEK/ERK.

Tyrosinase, the key enzyme of melanogenesis, is one of the glycoprotein which completes its maturation by processing *N*-glycosylation. Initial tyrosinase is glycosylated when translated polypeptide chain translocates into the endoplasmic reticulum (ER). After trimming by α-glucosidase, glycosylated tyrosinase interacts with chaperones and folds. Folded tyrosinase is completely matured via acquisition of two Cu^2+^ in Golgi and transported to melanosome [4]. Our data revealed that aerial part of *P. thunbergiana* reduces tyrosinase maturation (Fig. 10) by inhibiting α-glucosidase (Fig. 11).

UV radiation is a strong inducer of oxidative stress, contributing to skin pigmentation. Therefore, antioxidants can reduce melanogenesis [3, 19]. In this study, we detected antioxidant activity in the extracts of the aerial part of *P. thunbergiana* using DPPH assay, with the EtOAc fractions exhibiting stronger antioxidant properties than the aqueous fractions (data not shown). The EtOAc-soluble fractions showed remarkable scavenging effects compared to those of ascorbic acid, a well-known antioxidant [22]. The antioxidant activity could be regarded as one of the reasons that the aerial part of *P. thunbergiana* has anti-melanogenesis activity. Further study about antioxidant effect will be needed, because there are many mechanism linking antioxidant activity and melanin synthesis [27, 29].

**Fig. 16:**
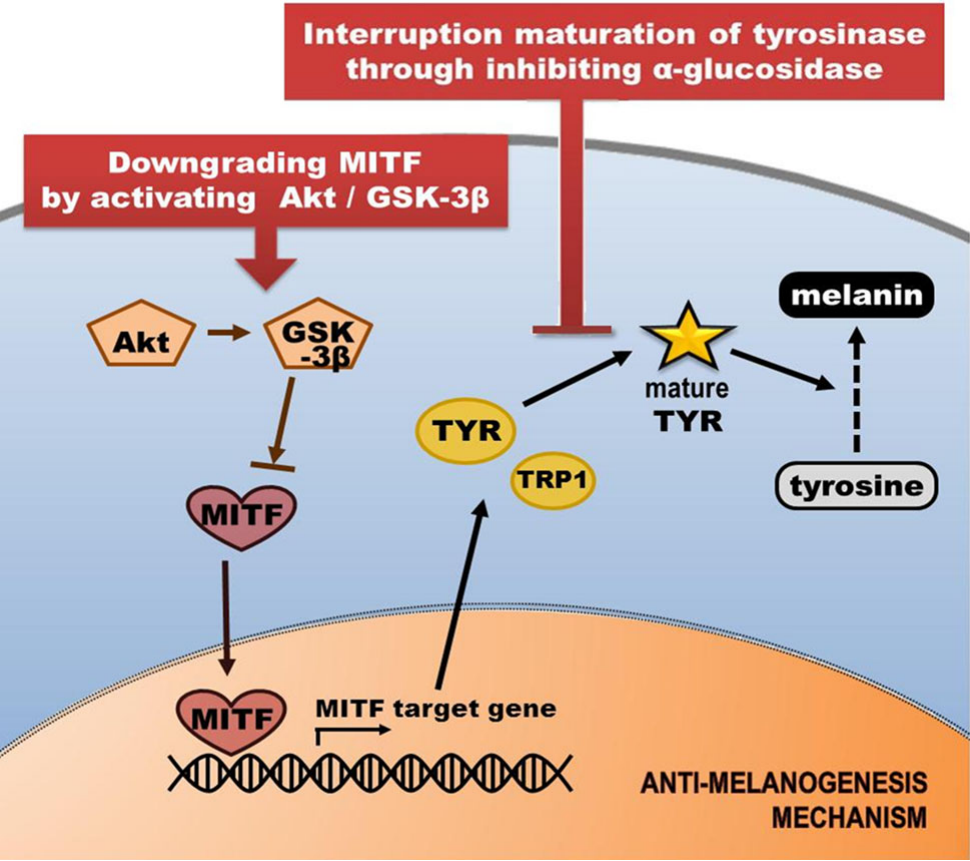
The multiple mechanisms of anti-melanogenesis effects of the aerial part of *P. thunbergiana.* The aerial part of *P. thunbergiana* has multi-action mediating inhibition of melanogenesis. The suppression in expression of tyrosinase and TRP1 is associated with MITF which is downgraded by Akt/GSK-3β signal. Maturation of tyrosinase is interrupted by inhibitory effect on α-glucosidase of the aerial part of *P. thunbergiana*. Antioxidant activity also inhibits melanogenesis

Taken together, the molecular and biological mechanisms underlying the inhibition of melanogenesis are mediated via complex pathways (Fig. 16). We confirmed that there are a lot of isoflavones in aerial part of *P. thunbergiana* (Table 1). The isoflavones are potent antioxidant agents and major active compounds of several Leguminosae plants (Fig. 3) [7, 21]. In practice, a study about the effect of genistein on melanogenesis by inhibitory effect on α-glucosidase has been reported [37]. Thus, it could be anticipated that the isoflavones are major components of aerial part of *P. thunbergiana* for anti-melanogenic effect. To define active compound, additional studies such as column chromatography and nuclear magnetic resonance (NMR) are going to be progressed.

Furthermore, we demonstrated that topical spread of cream contained extract of aerial part of *P. thunbergiana* can inhibit pigmentation in vivo study (Fig. 15). Application of aerial part of *P. thunbergiana* reduced UVB-induced-pigmentation, and the effect was more dramatical than kojic acid. In case of treatment of aerial part of *P. thunbergiana* 3 % contained cream, the skin lightness was higher than control that was not exposed to UVB. Furthermore, by Fontana–Masson staining study, it was identified that melanin distribution of epidermis induced by UVB was inhibited by aerial part of *P. thunbergiana* (Fig. 15c). These results of our study suggest that aerial part of *P. thunbergiana* can be used as a skin-whitening agent.
